# Widespread antimicrobial resistance among bacterial infections in a Rwandan referral hospital

**DOI:** 10.1371/journal.pone.0221121

**Published:** 2019-08-23

**Authors:** Tori Sutherland, Christophe Mpirimbanyi, Elie Nziyomaze, Jean-Paul Niyomugabo, Zack Niyonsenga, Claude Mambo Muvunyi, Ariel Mueller, Lisa M. Bebell, Theoneste Nkubana, Emile Musoni, Daniel Talmor, Jennifer Rickard, Elisabeth D. Riviello

**Affiliations:** 1 Department of Anesthesia, Critical Care and Pain Medicine, Beth Israel Deaconess Medical Center (BIDMC), Harvard Medical School, Boston, United States of America; 2 Department of Surgery, Kigali University Teaching Hospital, University of Rwanda, College of Medicine and Health Sciences, School of Medicine and Pharmacy, Kigali, Rwanda; 3 Department of Clinical Biology, Kigali University Teaching Hospital, University of Rwanda, College of Medicine and Health Sciences, School of Medicine and Pharmacy, Kigali, Rwanda; 4 Division of Infectious Diseases, Massachusetts General Hospital, MGH Global Health, and Harvard Medical School, Boston, United States of America; 5 Department of Pathology, Kigali University Teaching Hospital, Kigali, Rwanda; 6 Department of Surgery, University of Minnesota, Minneapolis, United States of America; 7 Division of Pulmonary, Critical Care and Sleep Medicine, Beth Israel Deaconess Medical Center, Harvard Medical School, Boston, United States of America; Rabin Medical Center, Beilinson Hospital, ISRAEL

## Abstract

**Background:**

Resistance among bacterial infections is increasingly well-documented in high-income countries; however, relatively little is known about bacterial antimicrobial resistance in low-income countries, where the burden of infections is high.

**Methods:**

We prospectively screened all adult inpatients at a referral hospital in Rwanda for suspected infection for seven months. Blood, urine, wound and sputum samples were cultured and tested for antibiotic susceptibility. We examined factors associated with resistance and compared hospital outcomes for participants with and without resistant isolates.

**Results:**

We screened 19,178 patient-days, and enrolled 647 unique participants with suspected infection. We obtained 942 culture specimens, of which 357 were culture-positive specimens. Of these positive specimens, 155 (43.4%) were wound, 83 (23.2%) urine, 64 (17.9%) blood, and 55 (15.4%) sputum. Gram-negative bacteria comprised 323 (88.7%) of all isolates. Of 241 Gram-negative isolates tested for ceftriaxone, 183 (75.9%) were resistant. Of 92 Gram-negative isolates tested for the extended spectrum beta-lactamase (ESBL) positive phenotype, 66 (71.7%) were ESBL positive phenotype. Transfer from another facility, recent surgery or antibiotic exposure, and hospital-acquired infection were each associated with resistance. Mortality was 19.6% for all enrolled participants.

**Conclusions:**

This is the first published prospective hospital-wide antibiogram of multiple specimen types from East Africa with ESBL testing. Our study suggests that low-resource settings with limited and inconsistent access to the full range of antibiotic classes may bear the highest burden of resistant infections. Hospital-acquired infections and recent antibiotic exposure are associated with a high proportion of resistant infections. Efforts to slow the development of resistance and supply effective antibiotics are urgently needed.

## Introduction

When accepting the Nobel Prize for the discovery of penicillin in 1945, Alexander Fleming cautioned that bacteria could become resistant to antibiotics that had revolutionized care for infected patients.[1] The World Health Organization (WHO) now credibly warns that we are headed toward a “post-antibiotic era” in which infections can no longer be treated with antibiotics.[2] Gram-negative bacterial infections resistant to broad-spectrum third-generation cephalosporins, carbapenems and now colistin have been identified.[3]

The world is divided into low, lower-middle, upper-middle and high income countries by the World Bank, based on gross national income per capita.[4] While it is clear that antimicrobial resistance (AMR) is a threat, most data on bacterial AMR are from high-income countries.[5] The resistance data that do arise from low-income countries (LICs) have been focused largely on tuberculosis, malaria, and human immunodeficiency virus (HIV).[6] In a recent international study examining bacterial infections among critically ill patients in 75 countries, only 1.1% of positive culture data originated from the African continent.[7] A review of the pediatric AMR literature from sub-Saharan Africa since 2005 identified only 18 articles, with evidence ranging from very-low to moderate quality.[8]

The scarce data that are available from LICs suggest that AMR is common.[9–12] A study of women with postpartum fever in Uganda found that among 25 blood and urine cultures with Gram-negative isolates, 80% were multi-drug resistant including cefepime-resistant.[13] In Rwanda, a retrospective study of cultures from patients in the internal medicine wards of a referral hospital revealed that almost a third of *Escherichia coli* isolates were resistant to one or more third-generation cephalosporins, and 82% of *Staphylococcus aureus* isolates were methicillin-resistant (MRSA).[14] A recent review of surgical-site infections in Rwanda found resistance to ceftriaxone in 53.3% of isolates and resistance to gentamicin in 92.6%.[15]

To determine the proportion of resistant isolates among patients with suspected infection in a LIC, we performed a prospective hospital-wide study of all adult patients with suspected infection in a referral hospital in Rwanda. We examined factors associated with third-generation cephalosporin resistance, and compared hospital length of stay and mortality outcomes for patients with and without resistant isolates.

## Methods

### Study oversight

The ethics committee of the University of Rwanda, College of Medicine and Health Sciences in Kigali, Rwanda, approved the study, as did the Committee on Clinical Investigations at Beth Israel Deaconess Medical Center (BIDMC) in Boston, Massachusetts. We asked for waiver of consent because collecting cultures in the setting of suspected infection is standard of care for the hospital (though it is often not achievable in clinical practice due to financial constraints.) The BIDMC IRB requested that we obtain verbal consent. Therefore, as approved by both ethics committees, patients with suspected infection were enrolled after verbal consent was obtained following a standard script in the patient’s primary language. Consent or refusal was documented on the patient screening enrollment log. Culture and sensitivity data from the study were made available to clinicians.

### Study design and setting

We designed this prospective observational study to characterize antimicrobial resistance among all adult inpatients in a referral hospital in Rwanda. The University Teaching Hospital of Kigali (KUTH) is a government-funded academic tertiary referral hospital in Kigali, Rwanda. It is one of three public referral hospitals in a country of approximately twelve million people. It has 560 total beds of which seven beds are intensive care unit (ICU) beds, and approximately 12,000 admissions each year. Rwanda has achieved almost universal heath insurance coverage;[16] most patients pay 10% of hospital charges, including laboratory studies, when in this public hospital. The hospital’s microbiology laboratory is managed by a senior technician and university microbiologist, who oversee eight technicians. It has refrigerators for plate storage and a generator for emergency power supply. The hospital is currently undergoing its first accreditation process with The Council for Health Service Accreditation of Southern Africa (COHSASA); in the initial assessment by COHSASA, the laboratory received a passing score.

### Study population and data collection

From January 25 through August 14, 2017, all adult patients (15 years or older, the criteria used to define adult ward admission at KUTH) were screened daily for inclusion criteria: temperature ≤35.0^o^ C or ≥38.0^o^ C and suspected infection, or undergoing surgery for an infectious process, regardless of temperature. Exclusion criteria included age<15, patient refusal to participate, and suspected viral or fungal source of infection, based on clinician judgment. Since many patients are unable to pay for laboratory studies, sample collection and testing costs were paid by the study. This allowed inclusion of data from all patients who met criteria for the study and consented, regardless of ability to pay.

Depending on the clinically suspected source of infection, blood (aerobic and anaerobic), urine, sputum, tracheal aspirate, wound, and/or surgical specimens were collected using standardized kits by the patient’s nurse, who sent specimens to the microbiology laboratory for processing. Urine samples were clean-catch or clean-catheterization samples collected after perineal cleansing. All surgical and open wound specimens were collected after sterile prep or saline cleaning. Tracheal aspirates were collected from intubated patients’ ventilation circuits. Blood, urine, and surgical sample collection was performed in an aseptic, sterile fashion; wound, sputum, and tracheal aspirate samples were non-sterile. Nurses were trained in standard sample collection, and re-trained throughout the study. A nurse educator provided additional training to locations in the hospital that were found to have higher than expected prevalence of contamination after an audit at the end of the pilot phase. We used standardized collection kits that included sterile alcohol wipes and single use gloves for collection.

Demographic and clinical data, including age, gender and co-morbidities, were collected from each participant’s chart and de-identified to protect privacy. Enrolled participants who developed a new fever or hypothermia and signs of infection after having been afebrile for >48 hours, and those who were taken back to the operating room for suspicion of a new infection, were re-enrolled with a new study identifier, with a crosswalk to link the two identifiers. Participants were followed through hospital discharge to determine length of stay and in-hospital mortality.

During the initial phase of the study, we recognized that a significant number of positive cultures in the laboratory had been drawn from patients who were not enrolled in our study because they had not been febrile, hypothermic, or undergone an infection source-control operation. We filed an amendment with both IRBs in March 2017. After approval, we added patients to the study who had positive bacterial cultures but had not been febrile on the ward and had not had an operation. We also began using the Center for Disease Control’s (CDC’s) two-step process for extended spectrum beta-lactamase (ESBL) confirmation[17] and added meropenem testing to the protocol to identify carbapenem resistance. All other protocols remained the same.

Active enrollment ended August 14, 2017 after the team reached our target of 300 positive cultures, and the remaining participants were followed until October 2017, when the last participant was discharged. Study data were entered into REDCap (Research Electronic Data Capture), a secure, web-based application, which was hosted at BIDMC.

### Laboratory methods

Blood samples were collected and incubated in BD BACTEC (Becton-Dickinson, Franklin Lakes, USA) bottles. Urine, wound and sputum cultures were collected in sterile containers; cutaneous wounds and purulent surgical sites were incubated in Amies vials. Blood cultures were directly incubated at 37°C and processed with the BACTEC system to identify bacteria. Samples positive for bacterial growth were sub-cultured on appropriate media guided by Gram stain results: Gram-positive cocci were plated on mannitol salt agar (MSA) and blood agar, and Gram-negative bacilli were plated on MacConkey agar and Xylose Lysine Deoxycholate agar (XLD) media. Additional identification of Gram-positive cocci species was done using catalase and coagulase tests. Gram-negative bacilli were identified by colony morphology. In addition, biochemical tests were performed, including triple sugar iron (TSI), motility indole urea (MIU), and citrate tests to identify and differentiate *Enterobacteriaceae* species.

Urine samples, after wet mount examination, were cultured on blood agar, cysteine lactose electrolyte-deficient (CLED), and MacConkey agar. The number of colonies were counted after 18–24 hours of incubation at 37°C, with maximum incubation 48 hours. Urinary specimens with > 10^5^ colony forming unit per milliliter (CFU/mL) urine were considered positive.[13, 18] For operative specimens, wound swabs and sputum specimens, the Gram stain morphology of principal pathogens dictated the selection of appropriate medium for culture, which was then incubated at 37°C for 24 hours. Similarly, identification of bacterial species was performed using a combination of colony morphology, growth characteristics, and biochemical tests.

Antibiotic susceptibility testing was performed by the Kirby Bauer disk diffusion method (S1 Table), per established laboratory protocol. Interpretation of the diameter of inhibition was done according to 2012 Clinical and Laboratory Standards Institute (CLSI) guidelines.[19]

For the second phase of the study, all laboratory technicians were trained in the CDC ESBL two-step screening and confirmation process[17] (S1 Table) We tested *Proteus spp*., *Klebsiella spp*. and *E*. *coli* isolates for ESBL production. Of note, we were unable to test all of these isolates for ESBL production due to supply chain limitations.

### Definitions

Participants were considered to have hospital-acquired infections if their fever or suspected infection occurred >48 hours after presentation to the hospital.[20] Comorbidity was defined as known diabetes, hypertension, tuberculosis, HIV, cancer, and/or severe malnutrition at the time of presentation.

A culture set refers to a set of cultures drawn at one time point, from all suspected sites of infection. A participant could have more than one set of cultures if they had a new fever and suspected infection, with an afebrile period of at least 48 hours between culture sets. Each culture set could include one or more specimens (wound/surgical specimen, urine, blood, and/or sputum/tracheal aspirate), and each specimen could grow one or more isolates.

The laboratory considered a blood culture specimen contaminated if only one of two samples was positive for coagulase- negative *Staphylococcus* (CnS). A urine culture was considered contaminated if more than three microbes were identified. Tracheal aspirates and wound swabs were considered contaminants if they produced CnS or Gram-positive rods.

We defined resistant isolates as Gram-negative bacteria that were resistant to at least one third or fourth generation cephalosporin (ceftriaxone, cefotaxime, ceftazidime, and/or cefepime), or met criteria for ESBL positive phenotype using the CDC definition.[17] We defined an isolate as MRSA if it was *S*. *aureus* resistant to cefoxitin, consistent with CDC guidelines, which were in turn based upon CLSI international standards.[19, 21]

### Statistical analysis

Data are presented as median (interquartile range) for continuous variables, mean (standard deviation) for discrete variables, and frequency (proportion) for categorical variables. Normality was assessed with the Shapiro-Wilk test. Statistical analysis was performed using SAS 9.4 (SAS Institute Inc., Cary, NC) with two-sided *p*-values < 0.05 considered statistically significant.

If a participant had multiple positive cultures in one culture set, only cultures with unique isolates were considered in the analysis. For example, if a participant had a blood culture positive for *E*. *coli* and a urine culture positive for *E*. *coli* and *Klebsiella spp*, the urine *E*. *coli* antibiogram was not included to prevent double-counting of isolates.

Because participants could have more than one culture set taken if they had new suspected infection after 48 hours of being afebrile, generalized estimating equations with robust variance estimators were used to assess for differences between culture type, accounting for within-subject correlation. In addition, a secondary analysis was performed with unique participants, using only the first set of cultures drawn for each participant (S2 Table).

We examined clinical and demographic characteristics of participants stratified by culture positivity and resistance: culture positive with a resistant isolate, culture positive but no resistant isolate, and culture negative.

Based on an historic hospital-wide mortality rate of approximately 10% and ICU mortality rate of 50%, we estimated a mortality rate of 30% among patients with antimicrobial resistant infections and 10% among patients with infections without resistance. Based on our clinical experience, we also estimated that two thirds of positive cultures would demonstrate resistance. Using these estimates, we found that we would need to collect 147 positive samples for the study to be adequately powered (power = 80%; alpha = 0.05) to detect a mortality difference between patients with resistant and sensitive isolates. To account for the possibility that mortality among patients with sensitive infections was higher than the hospital average (as high as 15%), we planned to collect a minimum of 279 positive cultures, with a target end point of 300 positive cultures.

## Results

Between January 25 and August 14, 2017, we screened 19,178 patient-days for suspected infection. We enrolled 647 unique study participants who had suspected infection and were cultured; only one patient who met study criteria declined enrollment.

From 647 unique participants, 762 sets of cultures were taken, totaling 942 specimens. Two of 942 specimens had missing results. Of 940 specimens with results, 357 (38.0%) were positive for bacterial growth, 489 (52.0%) were negative, and 94 (10.0%) were contaminated (Table 1). The 357 positive culture specimens came from 338 unique participants, with 364 unique isolates. Of 357 culture-positive specimens, 155 (43.4%) were wound specimens, 83 (23.2%) urine, 64 (17.9%) blood, and 55 (15.4%) sputum/tracheal aspirates.

**Table 1:** Culture positivity by specimen type.

	Positive (%) n = 357*	Negative (%) *n = 489*	Contaminated (%) *n = 94*	Total (%) n = 940**
**Specimen Type**				
Wound / surgical specimen	155 (43.4)	78 (16.0)	9 (9.6)	242 (25.8)
Urine	83 (23.2)	107 (21.9)	0 (0.0)	190 (20.2)
Blood	64 (17.9)	297 (60.7)	83 (88.3)	444 (47.2)
Sputum / Tracheal Aspirate	55 (15.4)	7 (1.4)	2 (2.1)	64 (6.8)

Blood, urine, and surgical sample collection was performed in an aseptic, sterile fashion; wound, sputum, and tracheal aspirate samples were non-sterile.

* 357 specimens were positive, and some had more than one type of colony. The 357 cultures represent 364 unique organisms and were obtained from 338 unique patients.

** A total of 942 cultures were obtained, however two had missing results for culture positivity and are not presented here (n = 940).

Gram-negative bacteria made up 323 (88.7%) of the 364 cultured isolates, and Gram-positive isolates were 41 (11.3%). *E*. *coli and Klebsiella* species comprised nearly two-thirds of all cultured isolates (Table 2). Gram-negative isolates were tested for cephalosporin susceptibility, and 183 of the 241 Gram-negative isolates tested for ceftriaxone resistance (75.9%) were ceftriaxone-resistant; 184 of 252 isolates tested (73.0%) were ceftazidime-resistant (Table 3). Of 92 *E*. *coli*, *Klebsiella spp*., *and Proteus spp*. formally tested for ESBL production, 66 (71.7%) met criteria for ESBL positive phenotype. Of the 250 Gram-negative isolates tested for ciprofloxacin susceptibility, 165 (66.0%) were resistant. Of the 296 Gram-negative isolates tested for resistance to carbapenems, 12 (4.0%) were resistant. Of 22 *S*. *aureus* isolates tested for cefoxitin susceptibility, seven (31.8%) met criteria for MRSA (Table 4).

**Table 2:** Distribution of organisms by culture type.

	Total *n = 364*	Blood *n = 66*	Urine *n = 74*	Tracheal Aspirate / Sputum *n = 62*	Wound / surgical specimen *n = 162*
**Gram Negative Organisms**					
* Escherichia coli*	139 (38.2)	18 (27.3)	52 (70.3)	10 (16.1)	59 (36.4)
* Klebsiella spp*.	91 (25.0)	15 (22.7)	15 (20.3)	19 (30.6)	42 (25.9)
* Proteus spp*.	26 (7.1)	1 (1.5)	3 (4.1)	5 (8.1)	17 (10.5)
* Acinetobacter spp*.	50 (13.7)	11 (16.7)	1 (1.4)	21 (33.9)	17 (10.5)
* Pseudomonas* spp.	15 (4.1)	0 (0.0)	2 (2.7)	7 (11.3)	6 (3.7)
* Salmonella* spp.	2 (0.5)	2 (3.0)	0 (0.0)	0 (0)	0 (0.0)
Gram Positive Organisms*					
* Staphylococcus aureus*	34 (9.3)	15 (22.7)	1 (1.4)	0 (0.0)	18 (11.1)
* *Coagulase-negative *Staphylococcus* spp.	2 (0.5)	2 (3.0)	0 (0)	0 (0.0)	0 (0)
* Streptococcus* spp.	5 (1.4)	2 (3.0)	0 (0)	0 (0.0)	3 (1.9)

Blood, urine, and surgical sample collection was performed in an aseptic, sterile fashion; wound, sputum, and tracheal aspirate samples were non-sterile.

*Testing was performed in order to identify *Enterococcus spp*; however, no positive cultures were observed.

**Table 3:** Proportion of gram-negative organisms resistant to tested antimicrobial agents.

Antimicrobial Agent	Total (%) n = 323*	*E. coli* *n = 139*	*Klebsiella spp.* *n = 91*	*Proteus spp.* *n = 26*	*Acinetobacter spp.* *n = 50*	*Pseudomonas spp.* *n = 15*	*Salmonella spp.* *n = 2*
**Amikacin,** n = 234	17 (7.3)	9 (9.7)	2 (2.7)	2 (9.1)	1 (3.0)	3 (25.0)	0 (0.0)
**Cefepime,** n = 183	98 (53.5)	41 (51.2)	32 (64.0)	7 (36.8)	13 (56.5)	4 (40.0)	1 (100.0)
**Ceftazidime,** n = 252	184 (73.0)	72 (66.7)	57 (82.6)	15 (68.2)	33 (86.8)	7 (46.7)	0 (0.0)
**Ceftriaxone,** n = 241	183 (75.9)	75 (67.0)	67 (85.9)	14 (63.6)	24 (92.3)	1 (100.0)	2 (100.0)
**Cefotaxime,** n = 227	166 (73.1)	81 (66.4)	65 (82.3)	16 (72.7)	2 (100.0)	0 (0.0)	2 (100.0)
**Cefuroxime,** n = 126	99 (78.6)	50 (68.5)	33 (89.2)	13 (100.0)	2 (100.0)	0 (0.0)	1 (100.0)
**Ciprofloxacin,** n = 250	165 (66.0)	73 (63.5)	52 (69.3)	16 (69.6)	17 (80.9)	5 (35.7)	2 (100.0)
**Trimethoprim/sulfamethoxazole (Cotrimoxazole),** n = 212	191 (90.1)	92 (93.9)	58 (90.6)	17 (85.0)	22 (78.6)	0 (0.0)	2 (100.0)
**Gentamicin,** n = 274	191 (69.7)	71 (59.7)	66 (82.5)	18 (81.8)	29 (78.4)	6 (40.0)	1 (100.0)
Carbapenem,**n = 296	12 (4.0)	0 (0)	4 (4.6)	0 (0.0)	3 (7.3)	5 (35.7)	0 (0.0)
**Piperacillin,** n = 49	37 (75.5)	12 (75.0)	6 (75.0)	3 (50.0)	12 (92.3)	3 (60.0)	1 (100.0)
**Piperacillin / Tazobactam,** n = 168	49 (29.2)	14 (19.7)	20 (40.8)	3 (15.8)	9 (47.4)	3 (30.0)	0 (0.0)
ESBL positive phenotype,***n = 92	66 (71.7)	37 (68.5)	24 (82.8)	5 (55.6)			

* The total number of unique Gram-negative organisms cultured was 323. Each organism was tested for resistance to some but not all antibiotics. See Supplemental S1 Table for laboratory protocols. The total n tested for each antibiotic is listed with each antibiotic name. The n tested by bacteria type is available upon request.

** This includes either Imipenem or Meropenem resistance.

*** *E. coli* (N = 54), *Klebsiella* (n = 29), and *Proteus* (n = 9) species were tested for ESBL using CDC guidelines when testing supplies were available; see methods for detailed protocol.

**Table 4:** Proportion of gram-positive organisms resistant to tested antimicrobial agents*.

Antimicrobial Agent	Total n = 41**	*S*. *aureus* *n = 34*	*Streptococcus spp* *n = 5*	*Coag negative staph spp* *n = 2*
**Ampicillin,** n = 22	15 (68.2)	13 (68.4)	2 (66.7)	0 (0.0)
Cefoxitin,***n = 22	7 (31.8)	7 (31.8)	0 (0.0)	0 (0.0)
**Cephalothin,** n = 6	1 (16.7)	1 (16.7)	0 (0.0)	0 (0.0)
**Ciprofloxacin,** n = 33	13 (39.4)	10 (34.5)	2 (66.7)	1 (100.0)
**Clindamycin,** n = 32	8 (25.0)	6 (23.1)	2 (40.0)	0 (0.0)
**Erythromycin,** n = 36	14 (38.9)	11 (36.7)	3 (60.0)	0 (0.0)
Carbapenem,****n = 24	3 (12.5)	3 (13.6)	0 (0.0)	0 (0.0)
**Linezolid,** n = 15	2 (13.3)	1 (8.3)	1 (33.3)	0 (0.0)
**Penicillin,** n = 30	27 (90.0)	24 (92.3)	3 (75.0)	0 (0.0)
**Tetracycline,** n = 36	18 (50.0)	14 (45.2)	4 (100.0)	0 (0.0)
**Vancomycin,** n = 37	5 (13.5)	3 (9.4)	2 (50.0)	0 (0.0)

* Testing was performed in order to identify *enterococcus spp*; however, no positive cultures were observed.

** The total number of unique Gram-positive organisms cultured was 41. Each organism was tested for resistance to some but not all antibiotics. See Supplemental S1 Table for laboratory protocols. The total n tested for each antibiotic is listed with each antibiotic name. The n tested by bacteria type is available upon request.

*** Cefoxitin resistance is an indicator of MRSA.

**** This includes either Imipenem or Meropenem resistance.

The median age of the 647 unique participants with suspected infection was 35 years (IQR 27, 51) (S2 Table). Of these, 22.1% had a known co-morbidity, including 10.5% with known HIV infection. In addition, 47.6% had undergone an operation within the 30 days prior to enrollment, and 65.2% had been given antibiotics within the 30 days prior to enrollment. Data on the type and duration of antibiotics prior to enrollment were not collected. More than half (57.8%) had hospital-acquired infections.

Age and gender were not associated with culture positivity or isolate resistance. Resistant isolates were significantly more likely to be present in participants transferred from another hospital (p = 0.01), those who had surgery within the prior 30 days (p<0.0001) and those who had been given antibiotics in the prior 30 days (p<0.0001). Isolates from hospital-acquired infections were also more likely to be antibiotic-resistant (p<0.0001) (Table 5).

**Table 5:** Patient characteristics stratified by culture positivity and resistance.

	All culture sets* n = 760	At least one positive culture with a resistant organism** n = 232	At least one positive culture but no resistant organisms** n = 106	All Cultures Negative *n = 424*	P-Value
Male gender, *n (%)*	423 (55.5)	129 (55.6)	50 (47.2)	244 (57.5)	0.11
Age***, *median (IQR)*	35 (27, 52)	35 (27, 52)	38 (30, 57)	35 (27, 51)	0.38
Patient lives outside Kigali***, *n (%)*^*2*^	437 (57.6)	155 (66.8)	50 (47.2)	232 (55.1)	0.02
Patient transferred from outside facility, *n (%)*	495 (65.0)	168 (72.4)	59 (55.7)	268 (63.2)	0.01
* *Outside facility length of stay (days), *median (IQR)*	2 (1, 5)	2 (1, 5)	1 (1, 4)	2 (1, 5)	0.60
Surgery within 30 days, *n (%)*	364 (47.8)	156 (67.2)	49 (46.2)	159 (37.5)	<0.0001
Antibiotic use in prior 30 days, *n (%)*	517 (67.8)	191 (82.3)	58 (54.7)	268 (63.2)	<0.0001
the -associated infection****, *n (%*)	481 (63.1)	188 (81.0)	54 (50.9)	239 (56.4)	<0.0001
Comorbidity*****	171 (22.4)	45 (19.4)	34 (32.1)	92 (21.7)	0.94
HIV	73 (9.6)	13 (5.6)	9 (8.5)	51 (12.0)	0.04
Temperature ≤ 35.0^o^ C or ≥ 38.0^o^ C	582 (76.4)	149 (64.2)	62 (58.5)	371 (87.5)	<0.0001
Heart rate > 100, *n (%)*	428 (56.2)	124 (53.4)	55 (51.9)	249 (58.7)	0.23
Systolic blood pressure < 90 mmHg, *n (%)*	42 (5.5)	6 (2.6)	6 (5.7)	30 (7.1)	0.02
On oxygen or oxygen saturation < 90%, *n (%)*	261 (34.2)	79 (34.0)	34 (32.1)	148 (34.9)	0.97
Intubated, *n (%)*	128 (16.8)	55 (23.7)	15 (14.1)	58 (13.7)	0.07

* This includes culture sets taken at different times from the same patient. A total of 762 culture sets were taken from 647 unique patients. Two culture sets were missing data, for a total of 760 culture sets to analyze. The within-subjects correlation was accounted for in the reported p-value by using generalized estimating equations with robust variance.

** Resistance is defined as any of the following: resistance to a third- or fourth-generation cephalosporin (ceftriaxone, cefotaxime, ceftazidime and/or cefepime), and/or confirmed ESBL-producer.

*** Missing data for age for seven culture sets. N = 755 for age. Missing data for three culture sets for whether a patient lives outside Kigali. N = 759 for this variable.

**** Defined as in the study hospital (KUTH) > 48 hours when culture set taken for suspected infection.

***** Includes patients who had any of the following documented co-morbidities: diabetes, hypertension, tuberculosis, cancer, and/or severe malnutrition.

Among non-hospital-acquired infections, isolates from participants on the obstetrics ward made up a significantly higher proportion of resistant isolates than other wards (31.8%; p = 0.03) (S3 Table).

**Fig 1:**
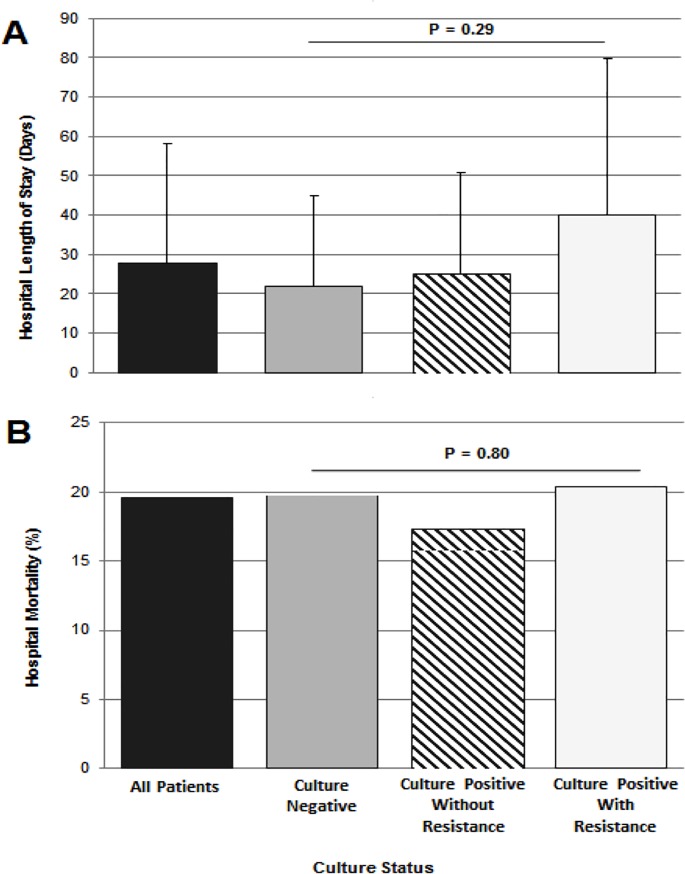
Hospital outcomes by culture status. Part (A) indicates hospital length of stay in days, and part (B) indicates hospital mortality rate, each stratified by culture type. Resistance is defined as any of the following: resistance to a third or fourth generation cephalosporin (ceftriaxone, cefotaxime, ceftazidime and/or cefepime), and/or confirmed ESBL positive phenotype.

The mean length of stay for participants with at least one resistant isolate was 40.0 days (SD 39.8) whereas it was 25.5 days (SD 25.3) for participants with positive cultures but no resistant isolates and 21.9 days (SD 23.0) for those with negative cultures *(*p *=* 0.29, Fig 1). Hospital mortality for all participants was 19.6% and did not differ significantly by culture positivity or resistance status *(*p *=* 0.80).

## Discussion

We prospectively performed bacterial cultures and antibiotic susceptibility testing on samples from all adult patients with suspected infection in a referral hospital in a LIC over a seven-month period. We identified an alarming rate of antimicrobial resistance in a population with limited and variable access to the full range of antibiotic classes. We found that nearly 90% of infections were due to Gram-negative isolates and that approximately 75% of these were resistant to third- or fourth-generation cephalosporins. Of isolates specifically tested for ESBL positive phenotype, 72% met criteria. Antimicrobial resistance was more common in patients who were transferred from another facility, had undergone recent surgery, had taken antibiotics recently, and those with hospital-acquired infections. In-hospital mortality among all participants with suspected infection was high, near 20%, but antimicrobial resistance was not associated with higher mortality.

Among patients with suspected infection, a higher proportion of bacterial isolates were Gram-negative than Gram-positive bacteria. One possible reason for this is the high proportion of positive cultures that came from wounds, which were most often operative samples for intra-abdominal infections. In an international study of predominantly high- and middle-income countries that found a more even distribution of Gram-positive and Gram-negative infections, 63% of isolates were from a respiratory source; our study had only 17% of specimens originating from sputum/tracheal aspirates.[7] In addition, variability by geographical location has been noted before, with Latin America demonstrating a significantly higher prevalence of Gram-negative bacterial infections than North America.[22, 23]

Our most striking finding was an extremely high proportion of third- and fourth-generation cephalosporin resistance. This finding is consistent with prior studies that suggest more resistance in poorer areas. An international study of 10,069 ICU patients in 84 almost entirely high- and middle-income countries found only 3% of Gram-negative infections to have an ESBL positive phenotype, though it is not clear what proportion of isolates were tested for ESBL positivity. [24] Another study examined over 5,000 isolates from 72 hospitals across the United States, and found ESBL resistance in 16% of *Klebsiella pneumoniae* isolates.[25] The same laboratory surveillance group[23] looked at over 5,000 Gram-negative isolates from 10 hospitals in four Latin American countries and found much higher rates of ESBL positive phenotypes: 60% of *Klebsiella spp*. isolates in Argentina, 50% in Brazil, 59% in Chile, and 33% in Mexico.[22] A recent study at another Rwandan referral hospital looked at intestinal carriage of ESBL positive *Enterobacteriaceae* and found a 50% carriage rate among patients on admission that rose to 65% on discharge, with 37% carriage among caregivers on admission, rising to 47% at time of discharge.[26]

A few studies in Africa suggest lower rates of antimicrobial resistance, but these studies’ methodologies were unlikely to capture the current rate of resistance. In a global point prevalence survey of antibiotic use, only 5% of patients in Africa who were receiving antibiotics and had a positive culture were receiving antibiotics for ESBL positive bacteria, though this result may have been driven by carbapenem scarcity rather than ESBL prevalence.[27] A retrospective review of wound cultures from 2004–2016 from six African countries found only 24% of *Enterobacteriaceae* isolates resistant to ceftriaxone.[12] However, the results were presented in aggregate for all twelve years, and it is likely that resistance has changed significantly over time. A study of blood samples drawn between 1998 and 2016 in a hospital in Malawi demonstrated increasing resistance over time, with the proportion of ESBL positive phenotype specimens reaching levels similar to what we found by 2016.[28]

Possible mechanisms for the high proportion of resistance seen in our study include those that have been hypothesized for all resource-constrained settings: widespread access to antibiotics within the community, use of antibiotics in agriculture, minimal regulation of the content of antibiotics, fewer resources for infection control measures within hospitals, and a lack of diagnostic modalities that enable de-escalation of antibiotics.[29] Some have also proposed a possible relationship between high humidity and high temperatures with certain Gram-negative bacterial infections that are prone to develop resistance.[22]

The impact of high rates of resistant isolates in a LIC setting is potentially devastating. Though ceftriaxone is one of the most commonly used antibiotics for serious infections in many settings, including Rwanda, we found 76% of Gram-negative isolates were ceftriaxone-resistant. We also found 72% of tested Gram-negative isolates to be ESBL positive phenotype. Currently, the only recommended treatments for ESBL positive infections are antibiotics from the carbapenem class (and in limited situations, cefepime).[30–33] Meropenem is the only carbapenem listed on the WHO essential medicine lists, and it is only on the complementary, not the core list.[34] In Rwanda, carbapenems are prescribed restrictively and are too expensive for most patients to afford. Although our study suggests a carbapenem would be appropriate empiric therapy among hospitalized patients in whom a Gram-negative infection is suspected, it is very rarely available even as targeted therapy. In our study, infection with a resistant isolate did not correlate with an increase in mortality. One possible explanation for this is that almost half of our positive culture specimens were wound cultures, for which source control may have reduced the need for antimicrobial therapy. It may also be that clinicians were able to use culture data to tailor antibiotic therapy to resistance patterns during the study. It is also possible that bacterial isolates acquiring resistance mutations did so at a cost to their virulence, resulting in eventual clearance of the infection by the participants’ own immune systems.[35]

Our study has several strengths. The laboratory is led by an experienced microbiologist and technician, and we were able to maintain a cold chain supply with stable electricity including a backup generator. This study is one of the first in East Africa that was able to perform confirmatory ESBL testing in specimens from multiple infection sites. In addition, we were able to analyze factors associated with resistance, compare risks and outcomes between hospital-acquired and non-hospital-acquired infections, and evaluate trends within different wards. All of these enable a more targeted approach to future interventions that might reduce infections with resistant isolates.

Our study also has several limitations. First, we did not collect data on duration and type of antibiotic use prior to enrollment. We also did not collect data on livestock exposure prior to admission, or chronic prophylactic antibiotic use (such as trimethoprim/sulfamethoxazole for participants living with HIV). This limited our ability to make inferences about some factors that may be contributing to resistance. Second, while we developed standard protocols for antibiotic testing, availability of antibiotic discs was limited early in the study. Thus, each isolate was not always tested for all recommended antibiotics per CLSI guidelines.[19] Unusual patterns of vancomycin-resistance were detected among isolated *Streptococci* samples; due to limited positivity, we are unable to definitively characterize this trend. Finally, our study was limited to one referral hospital in one LIC. It is not clear how generalizable the results are to other hospitals in other LICs; however, other studies suggest that our results may be applicable to many sub-Saharan African hospitals.[9–11, 13, 14, 26, 28] While our definitions of resistance to cephalosporins and MRSA do not include all isolates resistant to any antibiotic, they capture an important and clinically relevant subset of resistant isolates.

## Conclusion

Our study of adult inpatients with suspected infection in a Rwandan referral hospital found an alarming proportion of cephalosporin resistance among Gram-negative bacterial isolates. This suggests that, just as with multi-drug resistance in tuberculosis, low resource settings with the least access to alternative antibiotics may bear the highest burden of resistant infections.[36]

Our results imply an urgent need for pre-hospital and in-hospital interventions to slow the spread of antimicrobial resistance.

## Supporting information

S1 Data De-identified data. (XLSX)

S1 Table Laboratory protocol. (DOCX)

S2 Table Patient characteristics stratified by culture positivity and resistance, for first set of cultures taken. (DOCX)

S3 Table Patient location at time of infection stratified by culture status and when infection acquired. (DOCX)
