# Supplementary material for: Widespread antimicrobial resistance among bacterial infections in a Rwandan referral hospital
Source: PLoS One. 2019 Aug 23;14(8):e0221121. doi: 10.1371/journal.pone.0221121 (PMC6707788; doi:10.1371/journal.pone.0221121)
Supplement: S1 Table — (DOCX) [file pone.0221121.s002.docx]

**S1 Table. Laboratory protocol**

| **ORGANISM** | **ANTIBIOGRAM DISC 1** | **ANTIBIOGRAM DISC 2** | **ANTIBIOGRAM DISC 3** |
| --- | --- | --- | --- |
| **1. E. Coli, Klebsiella & Proteus**  **(Include 4^th^ disk for ESBL testing; see CDC protocol in text)** | 1. Gentamicin  2. Amikacin  3. Cotrimoxazole  4. Ciprofloxacin | 1. Amox/Clavulanic acid  2. Cefepime (in stock mid-Mar)  3. Ceftriaxone  4. Cefuroxime | 1. Piperacillin/tazobactum  2. Imipenem  3. Meropenem  **+4^th^ ESBL DISC** |
| **2. Citrobacter, Enterobacter (all other Enterobacter species members)** | 1. Gentamicin  2. Amikacin  3. Cotrimoxazole  4. Ciprofloxacin | 1. Amox/Clavulanic acid  2. Cefuroxime  3. Cefepime  4. Ceftriaxone | 1. Meropenem  2. Piperacillin/tazobactam  3. Imipenem |
| **3. Acinetobacter** | 1. Gentamicin  2. Ceftazidime  3. Imipenem  4. Amikacin | 1. Piperacillin/tazobactam  2. Cefepime  3. Cotrimoxazole  4. Ceftriaxone |  |
| **4. Pseudomonas** | 1. Ceftazidime  2. Gentamicin  3. Piperacillin/tazobactam  4. Amikacin | 1. Cefepime  2. Ciprofloxacin  3. Imipenem  4. Meropenem |  |
| **5. Staphylococcus aureus** | 1. Cefoxitin (MRSA)  2. Clindamycin  3. Cotrimoxazole  4. Vancomycin | 1. Ampicillin  2. Penicillin  3. Erythromycin  4. Tetracycline | 1. Linezolid  2. Imipenem  3. Amox/clavulanic acid  4. Ciprofloxacin |
| **6. Gram-positive; not specified above (ex. Enterococcus, streptococcus)** | 1. Penicillin  2. Ampicillin  3. Vancomycin  4. Linezolid | 1. Erythromycin  2. Amoxicillin  3. Ciprofloxacin  4. Tetracycline | 1. Cephalothin  2. Imipenem  3. Amox/clavulanic acid  4. Clindamycin |
| **7. Gram-negative; not specified above (ex. Providenica, Salmonella)** | 1. Cefuroxime  2. Ceftriaxone  3. Cefotaxime  4. Cefepime | 1. Piperacillin  2. Amikacin  3. Imipenem  4. Meropenem | 1. Ciprofloxacin  2. Cotrimoxazole  3. Amoxicillin/clavulanic acid |

Notes: Antibiotic susceptibility testing was performed by the Kirby Bauer disk diffusion method. A suspension from growth on a solid media plate was prepared by adding bacterial colonies into sterile distilled water until it approximated the same turbidity as the MacFarland turbidity standard 0.5. The resulting suspension was inoculated on Muller Hinton agar by using a sterile cotton swab. After this procedure, the antibiotic disks were added to the plate with at least 20 mm between each disk and subsequently incubated at 37°C for 18–24 hours; thereafter, interpretation of the diameter of inhibition was done according to 2012 Clinical and Laboratory Standards Institute (CLSI) guidelines. Quality control for the Kirby Bauer disk diffusion test was performed using three American Type Culture Collection (ATCC) strains: *Escherichia coli*ATCC 25922, *S. aureus*ATCC 25923, and *Pseudomonas*spp. ATCC 27853. Suspensions of the organisms were prepared as described above, and the inhibition diameter obtained was compared with the standard range expected for the ATCC strains.

The extended spectrum beta-lactamase (ESBL) plate contained four disks: ceftazidime with and without clavulanic acid and cefotaxime with and without clavulanic acid. Becton-Dickinson (BD) combination clavulanic acid disks were used. Screening was considered positive if the cefotaxime disk diffusion zone was <27mm or if the ceftazidime diffusion zone was <22mm. Confirmation of ESBL phenotype was documented if there was >5mm increase in diffusion zone when clavulanic acid was added to each antibiotic.

The following antibiotic disks were used: ampicillin, 10ug; cefepime, 30ug; ceftazidime, 30ug; ceftazidime/clavulanic acid 30/10ug; cefotaxime, 30ug; cefotaxime/clavulanic acid 30/10ug, ceftriaxone, 30ug; cephalothin, 30ug; cefoxitin, 30ug; cefuroxime, 30ug; ciprofloxacin, 5ug; trimethoprim/sulfamethoxazole (TMP/SMX), 23.75/1.25ug; amikacin, 30ug; amoxicillin/clavulanic acid (amox/clav), 20/10ug; clindamycin, 2ug; erythromycin, 10ug; gentamicin, 10ug; imipenem,10ug; linezolid, 30ug; meropenem, 10ug; penicillin, 10 units; piperacillin, 100ug; piperacillin/tazobactam, 100/10ug; vancomycin, 30ug; and tetracycline, 30ug.
