# Supplementary material for: Widespread antimicrobial resistance among bacterial infections in a Rwandan referral hospital
Source: PLoS One. 2019 Aug 23;14(8):e0221121. doi: 10.1371/journal.pone.0221121 (PMC6707788; doi:10.1371/journal.pone.0221121)
Supplement: S2 Table — (DOCX) [file pone.0221121.s003.docx]

**S2 Table. Patient characteristics stratified by culture positivity and resistance, for first set of cultures taken**

|  | **All first culture sets (unique patients)***  *N = 647* | **At least one positive culture with a resistant organism****  *N = 182* | **At least one positive culture but no resistant organisms****  *N = 91* | **All cultures negative**  *N = 374* | **P-Value** |
| --- | --- | --- | --- | --- | --- |
| Male gender*, n (%)* | 347 (53.6) | 98 (53.8) | 40 (44.0) | 209 (55.9) | 0.12 |
| Age***, *median (IQR)^1^* | 35 (27, 51) | 33 (26, 51) | 38 (29, 56) | 35 (26, 51) | 0.20 |
| Patient lives outside Kigali****, n (%)^2^* | 370 (57.4) | 124 (68.1) | 42 (46.1) | 204 (54.8) | 0.001 |
| Patient transferred from outside facility, *n (%)* | 414 (64.0) | 130 (71.4) | 50 (54.9) | 234 (62.6) | 0.02 |
| Outside facility length of stay (days), *median (IQR)* | 2 (1, 5) | 2 (1, 6) | 1 (1, 5) | 2 (1, 5) | 0.44 |
| Surgery within 30 days*, n (%)* | 308 (47.6) | 125 (68.7) | 42 (46.1) | 141 (37.7) | <0.0001 |
| Antibiotic use in prior 30 days*, n (%)* | 422 (65.2) | 147 (80.8) | 48 (52.7) | 227 (60.7) | <0.0001 |
| KUTH-associated infection | 374 (57.8) | 141 (77.5) | 41 (45.0) | 192 (51.3) | <0.0001 |
| Comorbidity***** | 143 (22.1) | 30 (16.5) | 28 (30.8) | 85 (22.7) | 0.02 |
| HIV | 68 (10.5) | 13 (7.1) | 8 (8.8) | 47 (12.6) | 0.12 |
| Temperature ≤ 35.0^o^ C or ≥ 38.0^o^ C | 497 (76.8) | 121 (66.5) | 52 (57.1) | 324 (86.6) | <0.0001 |
| Heart rate > 100, *n (%)* | 359 (55.5) | 98 (53.8) | 46 (50.5) | 215 (57.5) | 0.43 |
| Systolic blood pressure < 90 mmHg*, n (%)* | 37 (5.7) | 4 (2.2) | 6 (6.6) | 27 (7.2) | 0.05 |
| On oxygen or oxygen saturation < 90%, *n (%)* | 212 (32.8) | 62 (34.1) | 26 (28.6) | 124 (33.2) | 0.64 |
| Intubated, *n (%)* | 102 (15.8) | 43 (23.6) | 10 (11.0) | 49 (13.1) | 0.002 |

** This includes only the first culture set taken for each patient, so represents unique patients but excludes subsequent culture data for patients who had more than one set of cultures. A total of 762 culture sets were taken from 647 unique patients.*

*** Resistance is defined as any of the following: resistance to a third or fourth generation cephalosporin (ceftriaxone, cefotaxime, ceftazidime and/or cefepime), and/or confirmed ESBL-producer.*

**** Missing data for age for seven culture sets. N=640 for age. Missing data for two culture sets for whether a patient lives outside Kigali. N=645 for this variable.*

***** Defined as in the study hospital (KUTH) > 48 hours when culture set taken for suspected infection.*

****** Includes patients who had any of the following documented co-morbidities: diabetes, hypertension, tuberculosis, cancer, and/or severe malnutrition.*
