# Supplementary material for: Widespread antimicrobial resistance among bacterial infections in a Rwandan referral hospital
Source: PLoS One. 2019 Aug 23;14(8):e0221121. doi: 10.1371/journal.pone.0221121 (PMC6707788; doi:10.1371/journal.pone.0221121)
Supplement: S3 Table — (DOCX) [file pone.0221121.s004.docx]

**S3 Table.** **Patient Location at time of infection stratified by culture status and when infection acquired**

|  | **Non-Hospital Acquired Infections*** | | | **P-Value** | **Hospital Acquired Infections*** | | | **P-Value** |
| --- | --- | --- | --- | --- | --- | --- | --- | --- |
|  | **At least one positive culture with a resistant organism****  *N = 44**** | **At least one positive culture but no resistant organisms****  *N = 52**** | **All cultures negative**  *N = 184**** |  | **At least one positive culture with a resistant organism****  *N = 188**** | **At least one positive culture but no resistant organisms****  *N = 54**** | **All cultures negative**  *N = 238**** |  |
| **Patient Location at Time of Sample Collection** | | | | | | | | |
| Ward 3 (Internal Medicine) | 0 (0) | 2 (3.8) | 2 (1.1) | 0.27 | 13 (6.9) | 8 (14.8) | 44 (18.5) | 0.22 |
| Ward 4 (Internal Medicine) | 2 (4.5) | 1 (1.9) | 12 (6.5) | 0.23 | 18 (9.6) | 9 (16.7) | 23 (9.7) | 0.46 |
| Intensive Care Unit | 4 (9.1) | 5 (9.6) | 13 (7.1) | 0.72 | 55 (29.3) | 9 (16.7) | 31 (13.0) | 0.18 |
| Obstetrics | 14 (31.8) | 6 (11.5) | 18 (9.8) | 0.03 | 15 (8.0) | 4 (7.4) | 15 (6.3) | 0.75 |
| Emergency Department | 7 (15.9) | 16 (30.8) | 87 (47.3) | 0.0002 | 8 (4.3) | 4 (7.4) | 42 (17.6) | <0.0001 |
| Operating Room / Post-Anesthesia Care Unit | 13 (29.5) | 14 (26.9) | 36 (19.6) | 0.28 | 9 (4.8) | 2 (3.7) | 4 (1.7) | 0.18 |
| Neurosurgery | 0 (0.0) | 0 (0.0) | 0 (0.0) | --- | 9 (4.8) | 2 (3.7) | 8 (3.4) | 0.92 |
| Pavilion (private mixed ward) | 0 (0.0) | 1 (1.9) | 4 (2.2) | 0.62 | 3 (1.6) | 1 (1.8) | 4 (1.7) | 0.99 |
| Ward 1 (Surgery) | 2 (4.5) | 1 (1.9) | 2 (1.1) | 0.54 | 19 (10.1) | 2 (3.7) | 31 (13.0) | 0.03 |
| Ward 2 (Urology/Surgery) | 0 (0.0) | 2 (3.8) | 1 (0.5) | 0.15 | 12 (6.4) | 4 (7.4) | 20 (8.4) | 0.75 |
| Ward 7 (Burn/Orthopedics) | 2 (4.5) | 1 (1.9) | 1 (0.5) | 0.38 | 27 (14.4) | 8 (14.8) | 9 (3.8) | 0.003 |
| Ear/Nose/Throat Surgery | 0 (0.0) | 2 (3.8) | 1 (0.5) | 0.15 | 0 (0.0) | 0 (0.0) | 0 (0.0) | --- |
| Ward 6 (Tuberculosis) | 0 (0.0) | 1 (1.9) | 7 (3.8) | 0.65 | 0 (0.0) | 1 (1.8) | 7 (2.9) | 0.04 |

**. Non-hospital-acquired infections defined as culture sets taken within 48 hours of study hospital admission. Hospital-acquired infections defined as culture sets taken after patient had been in study hospital > 48 hours*

*** Resistance is defined as any of the following: resistance to a third or fourth generation cephalosporin (ceftriaxone, cefotaxime, ceftazidime and/or cefepime), and/or confirmed ESBL-producer.*

**** This includes culture sets taken at different times from the same patient. A total of 762 culture sets were taken from 647 unique patients. Two culture sets were missing data, for a total of 760 cultures to analyze. The within-subjects correlation was accounted for in the reported p-value by using generalized estimating equations with robust variance.*
